# Morphophysiological and Histopathological Effects of Ammonium Sulfate Fertilizer on *Aporrectodea trapezoides* (Dugès, 1828) Earthworm

**DOI:** 10.3390/life14091209

**Published:** 2024-09-23

**Authors:** Khaoula Aouaichia, Nedjoud Grara, Kamel Eddine Bazri, Edison Barbieri, Nedjma Mamine, Hadia Hemmami, Anna Capaldo, Luigi Rosati, Stefano Bellucci

**Affiliations:** 1 Laboratory Sciences and Technical Water and Environment, Department of Biology, Faculty of Natural Sciences and Life, Mohamed Cherif Messaadia University, P.O. Box 1553, Souk Ahras 41000, Algeria; aouaichia.khaoula@gmail.com; 2 Department of Biology, Faculty of Natural and Life Sciences and Earth and Universe Sciences, University of 8 Mai 1945 Guelma, P.O. Box 401, Guelma 24000, Algeria; 3 Laboratory of Ecology, Department of Plant Biology and Ecology, University Constantine 1, Constantine 25017, Algeria; k_bazri@yahoo.fr; 4 Instituto de Pesca, Governo do Estado de São Paulo, São Paulo 01027-000, Brazil; edisonbarbieri@yahoo.com.br; 5 Laboratory of Aquatic and Terrestrial Ecosystems, Department of Biology, Faculty of Natural Sciences and Life, Mohamed Cherif Messaadia University, P.O. Box 1553, Souk Ahras 41000, Algeria; nedjmamamine2022@gmail.com; 6 Department of Process Engineering and Petrochemical, Faculty of Technology, University of El Oued, El Oued 39000, Algeria; hemmami-hadia@univ-eloued.dz; 7 Renewable Energy Development Unit in Arid Zones (UDERZA), University of El Oued, El Oued 39000, Algeria; 8 Department of Biology, University of Naples Federico II, Via Cinthia, Edificio 7, 80126 Naples, Italy; acapaldo@unina.it (A.C.); luigi.rosati@unina.it (L.R.); 9 INFN-Laboratori Nazionali di Frascati, Via E. Fermi 54, 00044 Frascati, Italy; stefano.bellucci@lnf.infn.it

**Keywords:** ammonium sulfate, biomonitoring, chemical fertilizer, earthworm, ecotoxicology, histology

## Abstract

The present study used the adult earthworm *Aporrectodea trapezoides* as a bioindicator species to look into the possible dangers of ammonium sulfate (AS) fertilizer. Two complementary toxicity tests were conducted to determine the LC50values, growth rate inhibition, morphological alterations, and histopathological texture of worms. The lethality test included four increasing concentrations of AS fertilizer (ranging from 2500 to 7500 mg/kg of dry soil weight (d.w.)), while sub-lethal concentrations were based on 10%, 30%, 40%, and 50% of the 14-day median lethal concentration (LC50), with a control group included for both tests. The LC_(50)_ values for AS fertilizer were significantly higher at 7 days (4831.13 mg/kg d.w.) than at 14 days (2698.67 mg/kg d.w.) of exposure. Notably, earthworms exhibited significant growth rate inhibition under exposure to various concentrations and time durations (14/28 exposure days). Morphological alterations such as clitellar swelling, bloody lesions, whole body coiling and constriction, body strangulation, and fragmentation were accentuated steadily, with higher concentrations. Histopathological manifestations included severe injuries to the circular and longitudinal muscular layers, vacuolation, muscle layer atrophy, degradation of the chloragogenous tissue in the intestine, collapsed digestive epithelium of the pharynx with weak reserve inclusion, and fibrosis of blood vessels. These effects were primarily influenced by increasing concentrations of fertilizer and time exposure. The study highlights the strong relationship between concentration and exposure time responses and underscores the potential of *A. trapezoides* earthworms as valuable biological control agents against acidic ammonium sulfate fertilizer. Importantly, this research contributes to the use of such biomarkers in evaluating soil toxicity and the biological control of environmental risk assessment associated with chemical fertilizers.

## 1. Introduction

The massive augmentation of food demand, accompanied by continuous human population growth and the emerging mortal epidemic (COVID-19), recently made reaching global food demand sufficiency an impossible matter [1,2]. However, the only unique and faster solution that seems to be a decisive step is the usage of agrochemical products such as pesticides and chemical fertilizers on agricultural land as well as to increase crop yields. According to the International Fertilizer Industry Association [3], on average, 104,252 million tons of nitrogen, 40,522 million tons of phosphate, and 27,435 million tons of potassium are used each season worldwide. The haphazard usage of inorganic fertilizers and other agrochemicals in huge amounts affects the soil ecosystems adversely [4,5]. The overuse of nitrogenous fertilizers resulted in a cascade of environmental impacts and acidic conditions that threatened biodiversity worldwide [6,7], where the chemicals’ biomagnification in food chains reflected not only environmental pollution but also negative human health consequences [8]. In order to monitor the impact of soil pollutants, changes in soil structure, and agricultural practices, earthworms are considered suitable indicator species and the major soil macrofauna [9]. Due to their worthwhile role in agroecosystem and soil quality assessment, Jansirani et al. (2012) and Pelosi et al. (2014) [10,11] reported that earthworms are affected at all organism levels: changed individual behaviors, increased individual mortality, reduced fertility, and retardation of growth and reproduction. Using earthworms as a powerful ecotoxicological risk assessment tool to investigate the adverse effects of contaminants in soil ecosystems [12] through multiple developed tests depending on various toxicological endpoints such as growth, reproduction, and the avoidance behavior of earthworms [13,14]. Despite the importance of earthworms in the dynamic structure of soil, few data points have beenfound that associate earthworms with chemical fertilizers. In the literature, some researchers have demonstrated the positive effects of fertilizers on earthworms and their populations [15]. On the other hand, others highlighted their harmful impacts on soil organisms [16,17,18]. Thus, the effect of such agrochemicals on earthworms is poorly understood; further study is needed in order to clarify these discrepancies. Seamans et al. (2015) [19] reported that the field application of ammonium sulfate fertilizer limited only the abundance of *Aporrectodea* spp. and not *Lumbricus* spp. over two years. Yahyaabadi et al. (2018) [20] indicated a significant and sharp reduction in the survival and biomass of *L. terrestris* earthworms exposed to AS and DAP fertilizers compared to the control. Despite the wide use of inorganic fertilizers on agricultural land, very few studies have been conducted to date on the toxicity of these agrochemicals on earthworms, leaving many knowledge gaps that need to be addressed. Therefore, an understanding of the influence of these inorganic fertilizers on earthworms’ endpoints is extremely important for advancing sustainable agriculture and thereafter predicting soil pollution.

This is to ensure better consistency with well-intentioned data on the outcomes and facilitate comparing results obtained in different regions of the world. International guidelines were established by several organizations (OECD and ISO) to standardize the ecotoxicological methods conducted in laboratory conditions [13,21,22,23,24]. If earthworms are found to be important to agriculture, it will significantly enhance our understanding of the impacts of inorganic fertilizers on earthworms. This study hypothesizes that the effect of agrochemicals on earthworms is significant and that understanding it helps advance sustainable smart agriculture and soil pollution prediction. To fill the knowledge gaps in the literature, the present study aimed to (1) determine the median lethal concentrations (LC50) of AS fertilizer; (2) examine the ecotoxicological sublethal effects of this fertilizer on the endogeic earthworm *Aporrectodea trapezoides* endpoints, such as growth rate inhibition, morphological deterioration, and histopathological examination; (3) evaluate the relationship between different concentrations and time exposures of chemical fertilizer to replicate the natural conditions in the best possible way. To the best of our knowledge, this study constitutes the first baseline dataset to be used in ecotoxicology assessment, allowing the direct comparison ofLC50 values of the fertilizer mentioned above with the widely dominant earthworm in Algerian soil [25], and similar to other environmental settings, filling up some gaps in this subject’s knowledge.

## 2. Materials and Methods

### 2.1. Soil and Earthworm Collection

Soil and earthworm sampling were collected in an upland non-irrigated paddy field that had no record of agrochemical input (at least five years ago) from the Ain Ben Beida region, located in the northeast of Guelma province (36°28′11.05″ N, 7°25′42.89″ E) (i.e., Guelma/Algeria). Adult earthworms with a well-developed clitellum were randomly collected for a period of six months (November 2019 through April 2020), the most favorable period for soil macrofauna sampling in the region (favorable temperature and humidity). Based on its predominance in agricultural land, the *A. trapezoides* earthworm was adopted as the test specimen, with an average weight between 0.700 g and 1.800 g. All earthworms were kept in their native soil for 2 weeks of adaptation under laboratory conditions (20 ± 1 °C; photoperiods: 12 h light/12 h dark; pH: adjusted to 6.0 ± 0.5 and humidity: 35 °C) and fed with uncontaminated cow dung to provide food before the tests started. The soil sample was air-dried for 5 days, homogenized, and sieved through a 2 mm mesh to remove stones, roots, and other large particles prior to its use in the test. The main physicochemical characteristics of the soil (pH, K, Na, Mg, Ca, P, N, total carbon, electrical conductivity, and organic matter) were analyzed in the pedological analysis laboratory of the Algerian Fertilizer Company (Annaba’s FERTIAL) (Table 1). Knowledge of soil quality is therefore essential in order to ensure the validity of toxicity tests and their results.

### 2.2. Chemical Products

The inorganic fertilizer tested in these experiments is largely commercialized in Algeria: ammonium sulfate (21% N) fertilizer, which was purchased from Guelma’s regular fertilizer distributors (Profert brand) (i.e., Guelma/Algeria). AS fertilizer has a sustained action that ensures the gradual availability of nitrogen, limiting losses by leaching and preventing the occurrence of nitrogen deficiencies. AS is recommended for all crops and all soils.

The fertilizer solution that was applied was prepared in deionized water on the day of the experiments and used immediately.

### 2.3. Lethality Test

According to OECD Guideline 207 (1984) [21], acute toxicity experiments were carried out. The acute bioassay consisted of three replicates for each concentration, with 10 adult earthworms in each replicate. Afterward, they were placed in 5 L containers (25.5, 16, and 12.5 cm in height) with a softly perforated cover to maintain aeration and limit water loss. Based on a range-finding primary test (not recorded) that was performed with one replicate per treatment level, ammonium sulfate fertilizer was added at the following concentrations: 2500, 4500, 6500, and 7500 mg/kg of dry soil weight; in parallel, a control set was applied only with distilled water. About 1 kg of natural soil spiked with fertilizer solutions by simple pulverization was gently mixed, allowing homogeneous distribution of the fertilizer. Then, 250 mg (only on the first day of the experiment) of cow dung was added to each container in order to avoid starvation. The test was performed for a 14-day treatment period, and the number of dead earthworms per group was recorded on days 7 and 14 after the start of the experiment. Earthworms were considered dead when they did not react immediately to any mechanical stimulus in the anterior region.

### 2.4. Sublethality Test

The sublethal toxicity test was performed according to OECD (2004) guideline 222 [13] in triplicate with 10 adult earthworms in each replicate; 1 kg of natural soil substrate was introduced into 5 L plastic containers (25.5, 16, and 12.5 cm in height). The sublethal concentrations of AS fertilizer corresponded to 10%, 30%, 40%, and 50% of the 14-day median lethal concentration (LC_50_). In parallel, control series were also run with only distilled water. The natural soil substrate was spiked with the fertilizers’ solutions by simple pulverization. Throughout the exposures, earthworms were fed 7 g of fine cow dung (dried and rewetted to 80% moisture content) provided once a week during the 4th week of the exposure period.

#### 2.4.1. Growth Inhibition Rate

The biomass of tested earthworms was recorded on the 1st day, then after the 2nd and 4th weeks of the incubation period, earthworms were first removed, washed with distilled water to remove the mud attached to their bodies, dried, and weighed using an electro-balance, and after that, returned to the soil. To determine the worm’s growth inhibition rate (%) from various exposure periods and fertilizer concentrations, the following equation [26] was used:

GIn = (W_0_ − W_n_)/W_0_ × 100% (1)

where GIn (%) represents the growth inhibition rate, W_0_ represents the average weight of earthworms on the initial day, and W_n_ represents the average weight after n day of exposure.

#### 2.4.2. Morphohistological Study

Qualitative evaluation of morphological alterations and skin damage in earthworms was recorded only at the end of the sublethal toxicity test. For the histological study, control and treated earthworms were collected randomly on both the 14th and 28th days of the exposure period. Therefore, transverse sections from the preclitellar, clitellar, and postclitellar regions were prepared. The histological study was performed according to Hould and de Shawinigan’s (1984) [27] method. The samples were examined by optical microscope OPTICA AXIOM 7000 (Ponteranica, Italy) equipped with a digital camera (Optika ISview) and observed at a magnification of ×10.

### 2.5. Statistical Analyses

The normality of the data was verified using the Kolmogorov–Smirnov test, and the homogeneity of variances was checked by Levene’s test. The 7th and14th day LC50 values were estimated with Finney’s probit analysis through the concentration–response relationship of the *A. trapezoides* earthworm. Earthworms’ growth rates were determined in triplicate, and the data were analyzed using a two-way ANOVA, followed by a post hoc analysis using Fisher’s least significant difference (LSD) test, where *p* < 0.05 indicates a statistically significant difference, using SPSS software (version 20.0; SPSS, Chicago, IL, USA).

## 3. Results

### 3.1. Lethality Effects

**Figure 1:**
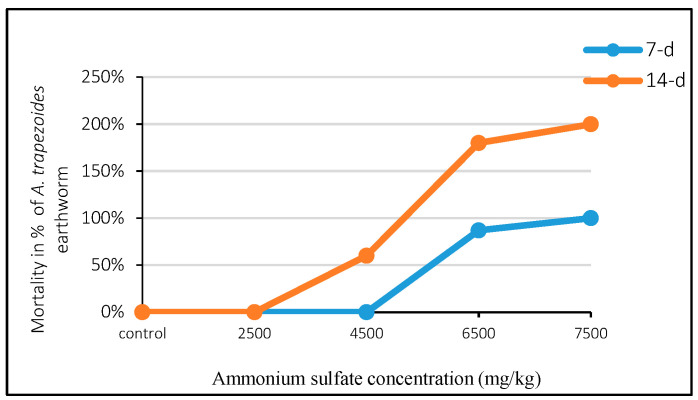
Mortality percentages of *A. trapezoides* earthworm under exposure to AS fertilizer in the acute toxicity test.

The results of this investigation demonstrate that the mortality of adult *A. trapezoides* earthworms exhibited significant variation in response to different concentrations of the tested inorganic fertilizer at various exposure times. The study was conducted with three replicates, including four concentrations of fertilizer and a control series (Figure 1). The mortality percentages recorded were as follows: 3%, 33%, 87%, and 100% when exposed to 2500, 4500, 6500, and 7500 mg/kg d.w. of AS fertilizer, respectively, compared to 53%, 60%, 93%, and 100% total mortality recorded at both 7 and 14 days, respectively.

The concentration–response test revealed no mortality in the control series (0 mg/kg) throughout the entire exposure period. Notably, there was a distinct variation in earthworm mortality across the different concentrations of AS in the three replicates. Additionally, all parameters of the acute toxicity test are listed in Table 2 and Table 3. The LC50 (lethal concentration) values for the ammonium sulfate fertilizer were relatively higher at 7 days (4831.13 mg/kg d.w.) compared to 14 days (2698.67 mg/kg d.w.) of exposure.

These results indicate that the mortality of adult *A. trapezoides* earthworms was influenced by the concentration of the tested inorganic fertilizer and the duration of exposure. Higher concentrations of AS and longer exposure times resulted in increased mortality. Furthermore, the LC50 values indicate that a concentration of 2698.67 mg/kg d.w. of AS caused 50% mortality in the earthworms after 14 days of exposure, while a higher concentration of 4831.13 mg/kg d.w. was required to achieve the same effect after 7 days of exposure.

These findings have important implications for the use of inorganic fertilizers, such as ammonium sulfate, and their impact on soil fauna, specifically earthworms. The high mortality observed at higher AS fertilizer concentrations and longer exposure periods suggests that indiscriminate use of these fertilizers may have negative effects on earthworm populations and, by extension, on soil health and associated ecological processes. Further studies are needed to better understand the underlying mechanisms and consequences of these effects.

### 3.2. Sublethality Effects

#### 3.2.1. Effects of Ammonium Sulfate on the Growth of Worms

The results of our study, analyzed using a two-way ANOVA test, indicate significant effects of ammonium sulfate (AS) concentrations and time exposures on the growth rates of earthworms. The main effect of AS concentrations was found to have a significant impact on lowering the growth rate of earthworms, with a statistically significant F-value (F4, 15 = 4.66; *p* = 0.031). Similarly, the main effect of time was also significant (F2, 15 = 4.75; *p* = 0.044).

The inhibitory effects of AS fertilizer on earthworm weight gain were prominently observed on the 14th and 28th days compared to the 1st day (*p* = 0.015). Furthermore, the biomass of worms decreased significantly following treatment with the 10% concentration compared to the control (*p* = 0.004). There was also a significant concentration relationship observed, as the 10% concentration led to a lower biomass compared to the 30% (*p* = 0.011), 40% (*p* = 0.023), and 50% (*p* = 0.017) concentrations.

The interaction between AS concentrations and time exposure had highly significant effects on the decrease in earthworm growth rate (F7, 15 = 88.55; *p* = 0.001). This indicates that the effect of AS concentrations on lowering the growth rate of earthworms was significantly greater on the 28th day compared to both the 14th day and the 1st day (control series).

**Figure 2:**
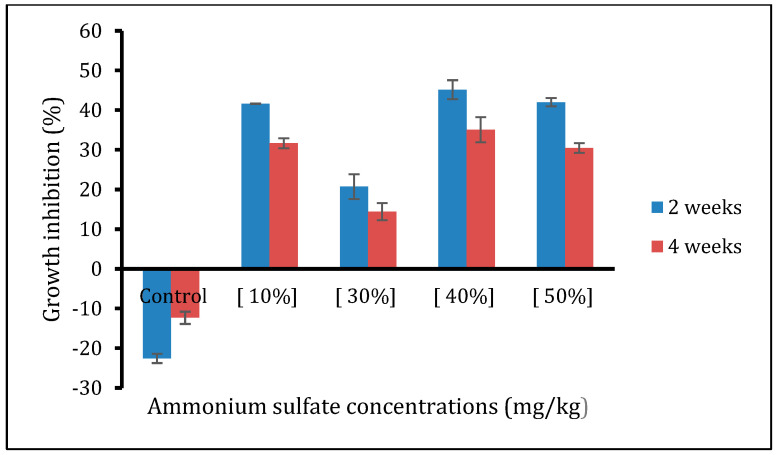
Growth inhibition rates (%) of *A. trapezoides* after exposure to different concentrations of ammonium sulfate fertilizer (mean ± SD, n = 3 repeats, with 10 individuals for each one).

The growth inhibition rates of *A. trapezoides* exposed to various concentrations of AS fertilizer, as presented in Figure 2, were determined using Shi et al.’s (2007) [26] equation. The control group exhibited negative growth inhibition rates at 2and 4weeks, indicating weight gain in earthworms. However, in all other concentrations of AS fertilizer and at all weeks, the growth inhibition rates were positive, indicating weight loss in earthworms. The growth inhibition rates varied significantly among different treatments and exposure times. Notably, the growth inhibition rates for all concentrations were higher in the 4th week compared to the 2nd week, indicating greater weight loss in earthworms at the later time point (Figure 2).

#### 3.2.2. Morphological Alterations 

**Figure 3:**
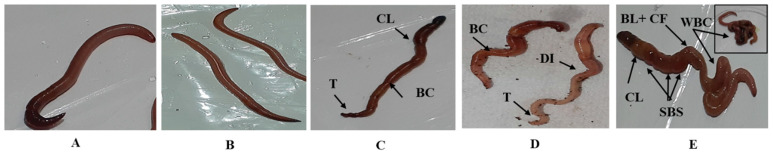
Morphological abnormalities in earthworm *A. trapezoides* exposed to different concentrations of AS fertilizer (**A**–**E**): (**A**) control, (**B**) 10% of 14 days’ LC50 of AS, (**C**) 30% of 14 days’ LC50 of AS, (**D**) 40% of 14 days’ LC50 of AS, and (**E**) 50% of 14 days’ LC50 of AS. CL—clitellar swelling; BC—body constriction; T—taper; SBS—successive body strangulation; DI—discoloration of the integument; BL—bloody lesions; CF—coelomic fluid; WBC—whole body coiling.

The results of our study indicate the occurrence of morphological changes in *A. trapezoides* earthworms exposed to ammonium sulfate (AS) fertilizer. The assessment of morphological changes was conducted on the 28th day of exposure. In the control series and at a low concentration of 10% AS fertilizer, intact bodies of the earthworms were observed. However, exposure to higher concentrations of AS fertilizer resulted in various morphological alterations. These included clitellar swelling, body constriction, tapering appearance, successive body strangulation, discoloration of the integument, bloody lesions with yellowish fluid evacuation, and whole-body coiling. The severity of these anomalies increased with higher concentrations of AS fertilizer, as shown in Figure 3.

Furthermore, the earthworms in the containers with higher concentrations of AS fertilizer exhibited altered behavior. They tended to remain at the bottom of the container, and a lower number of castings was observed compared to the control series. The presence of normal, intact bodies in the control series reaffirms the adaptability of the soil samples used in the toxicity tests. Unexpectedly, only at a low concentration of 10% AS fertilizer, undamaged and healthier worms were clearly observed.

#### 3.2.3. Histopathological Examination

**Figure 4:**
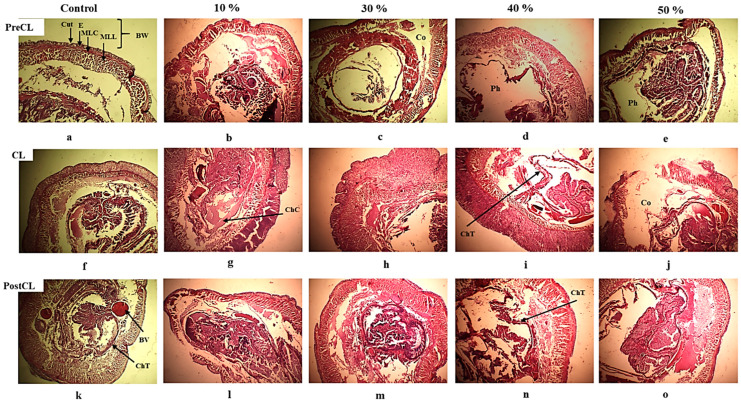
Histological cross-section of *A. trapezoides* after 14 days of exposure to AS fertilizer from low to high concentrations (magnification: ×10) passing through (**a**–**e**) PreCL—preclitellar region; (**f**–**j**) CL—clitellar region; and (**k**–**o**) PostCL—postclitellar region. Cut—cuticle; E—epidermis; MLC—circular muscle layer; MLL—longitudinal muscle layer; BW—body wall; Co—coelom (body cavity); BV—blood vessel; Ph—pharynx; ChT—chloragogen tissue; ChC—chloragogen cells.

**Figure 5:**
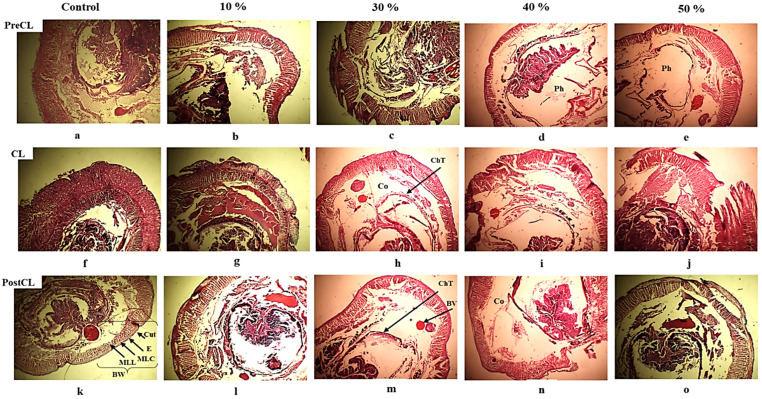
Histological cross-section of *A. trapezoides* after 28 days of exposure to AS fertilizer from low to high concentrations (magnification: ×10) passing through (**a**–**e**) PreCL, preclitellar region; (**f**–**j**) CL, clitellar region; and (**k**–**o**) PostCL, postclitellar region. Cut, cuticle; E, epidermis; MLC, circular muscle layer; MLL, longitudinal muscle layer; BW, body wall; Co, coelom (body cavity); BV, blood vessel; Ph, pharynx; ChT, chloragogen tissue.

The histopathological examination of transverse sections of control earthworms revealed a normal architecture of the body wall, including the skin, with continuous cuticle and epidermis membranes and naturally arranged circular and longitudinal muscle layers in all three regions. The epidermis was thinnest in the body wall, except for the clitellar region, where it was thicker. The pharynx, midgut, and chloragogenous tissue of the pre-clitellar, clitellar, and post-clitellar regions, respectively, showed intact architecture with clear outlines after both 14 and 28 days of exposure (Figure 4 and Figure 5a).

However, after 14 days of exposure to AS fertilizer, the earthworms exhibited increasingly severe injuries induced by higher concentrations in all regions.

In the preclitellar region, injuries such as necrotic cuticle (Cut) and epidermis (E) texture were seen. Loosely arranged circular and longitudinal muscular layers (MLC and MLL) were noticed, with emergent vacuoles and a focal profusion of blood plasma with spread cell debris turning around the pharynx. In parallel, the body wall was observed to be severely affected by external erosion and horizontal cleavage, accompanied by a degenerated texture of the digestive epithelium of the pharynx at the highest exposure concentration (Figure 4b–e).

In the clitellar region, severe necrotic lesions were observed intensifying on the internal and external body wall (BW) sides along with increasing concentrations, causing ruptured texture at high concentrations. Clear flattening of blood vessels (BVs) with deformed shapes was observed around the devastated texture of the atrophied chloragogenous tissue (ChT) of the midgut (Figure 4g–j).

Similarly, in the postclitellar region, necrotic lesions of the epidermis and cuticle were observed at all concentrations except in the control series. The body wall exhibited lateral distraction, and the muscular layers (MLC and MLL) were loosely arranged. Blood vessel flattening and chloragogen cell accumulation were noticed at the first concentration. As the AS concentrations increased, the dissemination of cell debris and blood vessel flattening intensified, leading to the devastation of chloragogenous tissue epithelium (Figure 4l–o).

At 28 days of exposure to AS fertilizer, the earthworms suffered even more drastic injuries, accentuated by increasing concentrations in all regions. 

The preclitellar region showed epidermal vacuoles, disarranged texture of MLL, and scrawny digestive epithelium of the pharynx at low concentration. Drastic necrotic lesions of the external body wall with proliferated cell debris and blood vessel flattening were observed, along with congested digestive epithelium of the pharynx. With increasing concentrations, the atrophied epithelium of the pharynx (with weak reserve inclusion) gradually decayed and scattered into the coelom (Co) (Figure 5b–e).

The clitellar region exhibited an external and internal erosive body wall with necrotic injuries that were intensified along with increasing concentrations, causing drastic vertical rupture with devastated integrity at the highest concentration. The deteriorated epithelium of the midgut tends to be congested by cell debris, then atrophied and degenerated (Figure 5g–j).

The postclitellar region displayed enlarged spaces between MLC and MLL, emerging cell debris proliferation, and many vacuoles. Blood vessel flattening and intestinal epithelium erosion intensified gradually with increasing concentration. The degenerated ChT epithelium with partial fissures of MLC and MLL, leading to huge vacuoles, cell debris dissemination, and poor reserve inclusion, was also observed at high concentrations (Figure 5l–o).

## 4. Discussion

The concentration–response tests found no mortality in the control series during all exposure times, indicating that the natural soil was adequate and considered valid to perform the toxicity tests according to the OECD (1984) guidelines [21]. At first, there were distinct variations in earthworm mortality (3%, 33%, 87%, and 100% compared to 53%, 60%, 93%, and 100% at 7 and 14 days, respectively), which increased steadily with increasing concentrations of AS fertilizer and exposure times. The most likely explanation for these negative results is, in fact, due to the acidified soil pH caused by ammonium sulfate fertilizer [28,29]. The low survivorship in very acid soils might depend on physiological disorders of earthworms, such as electrolyte and mucus secretion [30], that are affected by high exposure to H ions and inorganic Al, which could crowd in acidified soils [31,32,33]. Our findings corroborate the ideas of researchers [34,35], who ensured that nitrogenous fertilizers generate acidic conditions in the soil, which are fatal for earthworms. In a related study, Seamans et al. (2015) [19] found that the application of AS fertilizer in simulated runway experiments reduced only the field abundance of *Aporrectodea* spp. and not *Lumbricus* spp. over two years under six applications. This result could be explained by considering the endogeic ecological traits of *Aporrectodea* spp., which spends most of its lifetime in horizontal soil layers with a pH threshold of 4.7–5.7 [36], whereas the anecic *Lumbricus* spp. burrows vertically into the depth beyond the impact of AS fertilizer and withstands a wide range of pH levels (pH 3.6–5.0) [33]. Schnabel et al. (2020) [37] described similar responses with increasing concentrations of ammonium sulfate in water, which induced a stress response in western mosquito fish by increasing cortisol production. Contrary to expectations, some studies revealed that the applications of chemical fertilizers with nitrogen and phosphorous caused significant increases in earthworm abundance and biomass in oxisoil [38] and in calcic luvisol [39]. The most likely explanation for this positive correlation between earthworm biomass, abundance, and total nitrogen content in soil is the strong relationship that exists between phosphorous and nitrogen fertilizer, crop increase, and the feeding behavior of earthworms.

The results of the current test, as determined by Finney’s Probit analysis, reveal interesting findings regarding the toxicity of AS fertilizer on *A. trapezoides* earthworms. The LC50 values, representing the lethal concentrations of the fertilizer, were relatively higher at 7 days (4831.13 mg/kg d.w.) compared to 14 days (2698.67 mg/kg d.w.) of exposure. This indicates that AS fertilizer was found to be more toxic to *A. trapezoides* earthworms after 14 days of exposure compared to 7 days.

The observed lethal concentrations of AS fertilizer in our study demonstrated significant toxicological effects on the survival of earthworms. Surprisingly, these concentrations were found to be lower than the environmentally realistic dose (RAD = 600 kg/ha), according to the Profert data sheet. This means that the tested concentrations of AS fertilizer were below the legally allowed amount and within the ideal range recommended for agricultural use (recommended agricultural dose, RAD). These findings suggest that even at concentrations lower than the recommended dose, AS fertilizer can still have a notable toxic impact on *A. trapezoides* earthworms. It highlights the need for careful consideration and regulation of fertilizer application to mitigate potential adverse effects on soil fauna. The results also indicate the importance of assessing the toxicity of agricultural inputs to ensure environmentally safe practices and minimize potential harm to non-target organisms.

Further research is warranted to explore the underlying mechanisms of AS fertilizer toxicity on earthworms and to evaluate the long-term effects of exposure at environmentally realistic doses. Additionally, studies comparing the effects of AS fertilizer with other fertilizers or alternative agricultural practices could provide valuable insights into sustainable and ecological soil management strategies.

Our LC50 values of AS fertilizer results are comparable to recent results of Aouaichia et al. (2023) [40], where the LC50 values of potassium nitrate fertilizer on *A. trapezoides* were also relatively higher at 7 days (5530.43 mg/kg d.w.) than at 14 days (4955.70 mg/kg d.w.) of exposure. These findings revealed that the endogeic earthworm *A. trapezoides* was more sensitive to soil acidification caused by AS fertilizer, even at low concentrations, than soil salinization caused by potassium nitrate fertilizer. Our results are also in line with previous reviews. Frank et al. (1948) [41] reported that the LC50 of ammonium in rats is 3–4 g/kg, and Yamanaka et al. (1990) [42] reported it to be 2 g/kg in mice. Xu and Oldham (1997) [43] have also studied the lethal and sublethal effects of nitrogen fertilizer ammonium nitrate on common toads and found that the 96h and 168h LC50 values were 1704 mg/L and 1637 mg/L, respectively. This discrepancy in sensitivity could be explained by (1) various species with ecological differences and their food habitats (ecological groups) and (2) specific physiological characteristics of each species [44].

An inhibitory and significant effect on the growth rates and weight gain of *A. trapezoides* earthworms was noticed, along with increasing concentrations of AS fertilizer and longer exposure times (Figure 2). This emphasizes the potential adverse impact of AS fertilizer on earthworm populations and highlights the importance of considering its application in agricultural practices to minimize ecological harm. Further studies are necessary to elucidate the underlying mechanisms of AS-induced growth inhibition in earthworms and to explore potential mitigation strategies.

Sublethal effects such as earthworms’ growth inhibition rate are typical indicators for assessing the toxic effects of chemicals [26,45]. The two-way ANOVA test displayed greater significant inhibitory effects on weight gain at 28days than at 14days compared to the 1st day and control series. Moreover, the interaction between fertilizers’ concentrations and exposure times was found to be highly significant for earthworms’ growth rate decrease (*p* = 0.001). Indicating that the longer the exposure time, the more self-repairing function and anti-damage mechanisms of *A. trapezoides* decreased.

The noticeable earth worm biomass loss observed in the current study could be due to individual behavioral changes such as feeding rate, namely, the worms’ inability to directly ingest the acidified soil during exposure to AS fertilizer, which is similar to salinized soil caused by potassium nitrate fertilizer [40]. According to Curry (1994) [46], earthworms have been induced to starve rather than feed as a biological adaptation and niche problem in contaminated soils when the pollutant’s concentration exceeds the tolerable limits. The growth inhibition under exposure to toxic chemicals reflects chemical stress in earthworms [47,48], which is likely associated with the detoxification mechanism by which earthworms reduce food intake to avoid the disorder of membrane systems or the disruption of the cell membrane integrity from contaminants after exposure [49]. This behavior mainly contributes to the huge consumption of their energy reserves, such as protein, lipid, and glycogen, disturbing the normal metabolism and physiological function of bio-macromolecules in *A. trapezoids* [50,51].

Another recent study by Mekersi et al. (2022) [52] revealed that the growth rate, reproduction, and survival of the earthworm *A. trapezoids* were strongly inhibited by the high salinity levels, including the high acidic pH of olive mill wastewater (OMWW) and olive mill pomace (OMP), in addition to the higher level of phenolic compounds. Marco and Ortiz-Santaliestra (2009) [53] claimed that the hazardous changes in earthworms’ individual growth, development, and behavior due to nitrogenous compounds might indirectly translate into reduced population viability. In the same vein, other recent results [54,55] affirm that exposure times and ammonium hydroxide’s increasing concentrations affect earthworms’ biomass adversely. A variety of chemicals show different levels of toxicity potential in different animal models, indicating that no single chemical shows the same toxicity potential.

The observed morphological alterations in *A. trapezoides* earthworms exposed to AS fertilizer highlight the potential negative effects of this chemical on their well-being. The increasing severity of morphological changes (including clitellar swelling, body constriction, tapering appearance, successive body strangulation, discoloration of the integument, bloody lesions with yellowish fluid evacuation, and whole-body coiling) with higher concentrations of AS fertilizer suggests a dose-dependent response. Likewise, *A. trapezoides* displayed almost similar abnormal symptoms under exposure to potassium nitrate fertilizer [40]. These findings underscore the importance of considering the indirect effects of chemical substances or xenobiotics on the behavior and morphology of earthworms, as these effects can have consequences for the functions of soil ecosystems.

It is worth noting that the morphological alterations observed in this study provide valuable insights into the sublethal effects of AS fertilizer on earthworms. However, further research is needed to understand the underlying mechanisms and long-term consequences of these morphological changes. Additionally, assessing the recovery potential of earthworms after exposure to AS fertilizer and investigating potential mitigation measures would contribute to developing more sustainable soil management practices. These results are in line with previous studies by Bouazdia (2020) [56], who demonstrated the morpho toxicological effect of Decis insecticide on *A. caliginosa* after 4weeks of exposure. In the same vein, Singh et al. (2019) [57] obtained almost comparable morphological changes in worms exposed to pesticide triazophos that were accentuated mainly in the case of mixing with deltamethrin. Furthermore, *Metaphire posthuma* exposed to phorate suffered from winding, wall disruption with the appearance of internal glandular cell mass, and disintegration of circular and longitudinal muscles that led to fragmentation of the body [58]. The behavioral impairments like swelling, coiling, and curling despite no feeding; were found in the endogeic *A. trapezoids* and are undoubtedly attributed to the sensitivity of this species to high soil acidity conditions. The main reason for earthworms’ morphological alterations despite their previous starvation (discussed earlier in the work of Curry (1994) [46]) could only be explained by the infiltration of contaminants through dermal contact (the skin route). Overall consequences revealed that there was a wide variation in the toxicity of AS fertilizer tested on *A. trapezoides* earthworms compared to the control series. Our results provide new insights in a toxicological context and reveal that the endogeic earthworm *A. trapezoides* is more sensitive to soil acidification caused by AS fertilizer, even at low concentrations. Moreover, the LC50 values decreased during the 14-day exposure period, revealing a higher, or double, toxicity of AS fertilizer at 14 than at 7 days. Thereby, the toxicity of fertilizer displayed a strong concentration–time–response relationship.

The histopathological findings indicate that exposure to AS fertilizer caused severe damage to *A. trapezoides* earthworms. The injuries included necrosis, erosion, disarrangement, and depletion of various tissues in different body regions. The severity of the damage increased with higher concentrations and longer exposure durations. These observations highlight the detrimental effects of AS fertilizer on the structural integrity and overall health of earthworms. Further research is needed to understand the underlying mechanisms of these histopathological changes and their implications for earthworm populations and the soil ecosystem.

Ecotoxicology studies are performed to reveal the basic mechanisms by which pollutants may disrupt the normal physiological state of biological systems and to prevent the negative consequences arising from them [59]. Histopathology is a popular tool to assess the environmental impact of possible exogenous contaminants on various living organisms, including earthworms [60]. The histopathological changes of earthworms are increasingly used to monitor soil pollutants in the environment. Considering the key role played by the muscle layers in the body wall of earthworms, it is evident that any injurious effect on muscular layers contributes significantly to losing their displacement ability and ecological functions. It is well documented that the intestinal epithelium and the epidermis of earthworms are the main barrier tissues to meet soil pollutants through direct contact, digestion, and absorption. It has been reported that any damage to the epithelium layer hinders the selective permeability of any pollutants in the soil that may easily enter the organism without endocytosis [61,62]. Thus, any significant lesions may occur on heavily damaged tissue, eventually leading to death [60].

The earthworm *A. trapezoids* exposed to 10%, 30%, 40%, and 50% of 14 days’ LC50 value of ammonium sulfate did not exhibit the distortion in their normal histological architecture on the 14th day, even on the 28th day of the exposure period. The histopathological examination revealed that the exposure to gradually increased concentrations of AS fertilizer affected not only the epidermis but also the circular and longitudinal muscular layers, resulting in serious injuries with vacuolation that could seriously impair the locomotor ability of the worm. Muscle layers atrophy mainly due to myofiber decline, which results in high protein deterioration [63,64]. The unaccustomed muscle contraction due to stress might lead to an acidic soil pH, resulting in the loss of cellular integrity. This demolition process can be induced by chronic inflammation and acute metabolic changes [65]. In addition to focal internal damage, a degradation of the slender chloragogenous tissue of the intestine, collapsed digestive epithelium of the pharynx with weak reserve inclusion, fibrosis of the blood vessel with shape deformation, and an accumulation of cell debris in the coelom could seriously impair their ecological functions. Clitellar damage could impair earthworms’ cocoon production and other reproductive functions. These damages are accentuated mainly with increasing concentration and time exposure. The control earthworms showed normal architecture of the body wall and intact blood vessels with a complete structure of the pharynx and intestine.

Reports are available on the effects of diverse groups of chemicals on the histology of different earthworm species, which are more or less similar to the results obtained in this study. Comparable histopathological responses were found under exposure to organic pollutants [66,67,68]. The most common responses were disintegration of the cuticular membrane and the ectoderm layers, damage to the circular and longitudinal muscles due to necrosis, deformation of chloragogenous cells, and tissue erosion, the latter usually leading to body fragmentation [68,69,70,71,72,73]. Qi et al. (2018) [74] found that a novel neonicotinoid insecticide, cycloxaprid, induced more damage to the earthworm gut, nerves, epidermis, and atrophy of the longitudinal muscle layer. The exposure to other pollutants, such as pesticides, metals, and nanoparticles, showed dilatation and degradation of the intestinal tract [40,75,76]. High concentrations of pesticides could cause significant damage to the muscle and intestinal epithelium of the tropical earthworms *G. tuberosus* and *E. eugeniae* [77,78]. Loss of structural integrity and vacuolated chloragogenous tissue were observed in *Lumbricus terrestris* isolated from heavy metal-contaminated soil [67]. With increasing concentrations of nickel (Ni), the circular and longitudinal muscle layers were severely collapsed, and the structural integrity was completely altered. The intestinal epithelium also displayed severe signs of enlargement of the intestinal tract and a decrease in the body cavity. Considerable damage to the chloragogenous tissue, vacuolation of the epithelium, an increase in the number of pyknotic cells, the loss of nuclei from the epithelial cells, and disorganized muscle fibers in the earthworm *Nsukkadrilusmbae* exposed to sublethal doses of the herbicide atrazine have been reported [79]. Stanley and Ochulor (2014) [80] observed disrupted circular and longitudinal muscles, cytolysis, tissue vacuolization, cell necrosis, and cuticular degeneration in the same species exposed to sublethal concentrations of glyphosate. Deleterious histopathological changes in various species of earthworms because of toxicity due to heavy metals, hydrocarbons, mines, and organic wastes have also been observed [61,81,82,83,84,85].

To the best of our knowledge, there is no histopathological data regarding the toxicity of AS fertilizer for soil invertebrates, particularly in the three regions (preclitellar, clitellar, and postclitellar regions). Indeed, an early study and research report by Samal et al. (2017, 2019, and 2020) [78,86,87] revealed the histopathological transverse sections of *Drawida willsi* and *Lampito mauritii* earthworms exposed to urea, phosphogypsum (PG), and paper mill sludge (PMS). Moreover, they studied the single effects of the three above-mentioned agrochemicals with two other organophosphate pesticides, monocrotophos and glyphosate, on *Eudrilus eugeniae* earthworms and, in addition, the effects of the two last pesticides, on *Drawida willsi* and *Lampito mauritii* earthworms, respectively. In the same three regions (preclitellar, clitellar, and postclitellar regions) as our study, they found considerable lesions and skin undulation, disintegration of connective tissue, vacuolation of the dermis, and muscle disorganization in earthworms. However, in our study, we have examined not only the body wall but also the internal ones (pharynx, midgut, blood vessels, and ChT) of the entire worm.

Collectively, the adverse effects of the outer body wall and the intestine may indeed have a deleterious reflection on the health of earthworms because they delay the normal functioning of these tissues and therefore their homeostasis. The intestinal barrier is considered a key factor leading to the pathogenesis of intestinal bacteria [88]. It is also possible that the acidic pH of AS fertilizer decreases the content of 2-hexyl-5-ethyl-furan-3-sulfonic acid (HEFS), a unique surfactant found in the earthworm gut, which might play a vital role in stabilizing the intestinal cell membrane [89]. In addition, a stable and healthy gut microbiota is very important for earthworm health [90]. Additionally, a study reported that tissue injury affects the bioenergetics of earthworms, which ultimately disturbs the overall energy budget, leaving less energy for physiological processes like growth and reproduction [91]. Any cell death or necrosis that is not rapidly repaired usually produces failures in osmotic regulation [69]. According to Bowen and Lochshin (1981) [92], cell death refers to heterogeneous mechanisms in structure and biological function. Necrosis is characterized by cytoplasmic swelling, mitochondrial damage, and an excessive loss in osmotic regulation caused by the loss of cellular energy reserves. In light of the above data and literature information, we can conclude that, as a mechanism to prevent osmotic failures, earthworms have a large regeneration capacity. In cases of tissue damage, the chloragogen cells are able to migrate to the wound or lost tissue and regenerate it [72,93,94,95]. Chloragogen tissues are food reserves and may accumulate great amounts of Ca, P, S, Cl, Zn, K, and Fe [96,97,98,99]. These elements are released into body fluids to increase osmoregulation during stress [100]. However, our 28th-day results affirmed the inability of the *A. trapezoides* earthworm to regenerate the lost tissue and deteriorated texture after 14 days. Alterations in chloragogen cell activity are likely to be responsible for the observed impairment in enzymatic activities (i.e., ChE, LDH, and ALP).They can be considered precursors of lethal and sublethal effects.

To our knowledge, this is the first study in which the effects of AS fertilizer on the earthworm *A. trapezoids* (mortality, biomass, morphology, and histological endpoints) were investigated. Furthermore, this study could be used as an earlier warning signal of inorganic fertilizer contamination, thereafter encouraging earthworm endpoints to be among the standardized tests to assess the chemical fertilizer’s pollution individually.

## 5. Conclusions

Overall, this study demonstrates awareness of the systematic interaction between ammonium sulfate fertilizer and the earthworm *A. trapezoids,* shedding new light on the ecotoxicological, morphophysiological, and histopathological contexts. Severe histopathological textures in the three above-mentioned regions of the worm emphasize the sensitivity of *A. trapezoids* earthworms to chemical fertilizers. Such conditions may pose a serious threat to these organisms’ lives. It can be concluded that these approaches could be used as significant biomarkers for early warning and diagnosis of soil pollution. Simultaneously, *A. trapezoides* species might be used as a terrestrial pollution bioindicator to assess the ecological risks associated with the application of chemical fertilizers. The results indicate the importance of assessing the toxicity of agricultural inputs to ensure environmentally safe practices and minimize potential harm to non-target organisms by knowing their LC 50 value and aiming for careful consideration and regulation of fertilizer application. Regarding the negative effects of chemical fertilizers on earthworms, further studies would be worthwhile as future research in order to investigate other toxic effects from a more in-depth knowledge perspective, i.e., oxidative stress biomarkers (GSTs, GPx, CAT, MDA, GSH, SOD, POD, LDH, and CYP 450), bimolecular level, DNA damage, and innate immunity. Advancing towards higher effectiveness of ecotoxicological assessments, these results reflect meaningful information concerning the risks of applying such agrochemicals to soil quality and biodiversity.

## Figures and Tables

**Table 1:** The physicochemical characteristics of the soil.

Properties	Results
pH	7.34 ± 0.01
K	0.17 ± 0.2 (meq/100 g)
Na	1.57 ± 0.05 (meq/100 g)
Mg	2.68 ± 0.34 (meq/100 g)
Ca	34.93 ± 0.15 (meq/100 g)
P	1.80 ± 0.23 ppm
N	0.150 ± 0.65%
Total carbon	7.5 ± 0.04%
Electrical conductivity	0.16 ± 0.54 mS/cm
Organic matter	2.060 ± 0.08%
Texture	Sand 12%; silt 76%; Clay 12%

**Table 2:** Lethal concentrations (LC _10_, LC_25_,LC_50_, LC_90_) of AS fertilizer after 7days of exposure.

Exposure Conc. mg/kg	Total Test Worms	No. of Dead Worms Replicate			Total No. of Dead Worms	% Mortality	LC_10_ (mg/kg)	LC_25_ (mg/kg)	LC_50_ (mg/kg)	LC_90_ (mg/kg)
		1	2	3						
0	30	0	0	0	0	0	3533.44	4131.65	4831.13	6605.42
2500	30	0	0	1	1	3.33				
4500	30	3	4	3	10	33.33				
6500	30	6	10	10	26	86.66				
7500	30	10	10	10	30	100				

During the Probit analysis, the chi-square value, χ^2^, was 3.525, and no heterogeneity factor was observed at 95% confidence limits. The coefficient of determination value, R^2^, was 0.986.

**Table 3:** Lethal concentrations (LC _10_, LC_25_, LC_50_, LC_90_) of AS fertilizer after 14days of exposure.

Exposure Conc. mg/kg	Total Test Worms	No. of Dead Worms Replicate			Total No. of Dead Worms	% Mortality	LC_10_ (mg/kg)	LC_25_ (mg/kg)	LC_50_ (mg/kg)	LC_90_ (mg/kg)
		1	2	3						
0	30	0	0	0	0	0	1128.27	1744.94	2698.67	6454.84
2500	30	5	6	5	16	53.3				
4500	30	5	7	6	18	60				
6500	30	8	10	10	28	93				
7500	30	10	10	10	30	100				

During the Probit analysis, the chi-square value, χ^2^, was 9.412, and no heterogeneity factor was observed at 95% confidence limits. The coefficient of determination value, R^2^, was 0.721.

## Data Availability

It was integrated as document Word by email.
